# Venous Air/Gas Embolism During Surgical Hysteroscopy: An Underestimated, Potentially Lethal Complication

**DOI:** 10.7759/cureus.114578

**Published:** 2026-08-15

**Authors:** Eunkyung Choi, Ji-yong Yeom, Min Gang Kwon, Gaeul Hur

**Affiliations:** 1 Department of Anesthesiology and Pain Medicine, Yeungnam University College of Medicine, Daegu, KOR

**Keywords:** anesthesia, case report, hemodynamic instability, surgical hysteroscopy, transthoracic echocardiography, venous air/gas embolism

## Abstract

Hysteroscopic surgery is generally considered low risk; however, venous air/gas embolism is a potentially lethal complication with high mortality in severe cases. We summarize the pathophysiology and clinical features to support timely emergency management. A 46-year-old woman with a uterine myoma underwent hysteroscopic surgery under general anesthesia. Approximately 50 minutes after the start of the procedure, a sudden decrease in end-tidal CO₂, bradycardia, hypotension, and ST-segment elevation were observed. Transthoracic echocardiography revealed air bubbles in the right ventricle, confirming venous air/gas embolism. The procedure was discontinued, and immediate supportive management, including hemodynamic stabilization and intensive care monitoring, was initiated. The patient recovered without sequelae and was discharged on postoperative day 7. This case highlights an unusual cardiovascular presentation of venous air/gas embolism, with profound bradycardia and marked ST-segment elevation mimicking acute coronary syndrome, and emphasizes the importance of rapid diagnosis and intervention.

## Introduction

Hysteroscopy has gained increasing popularity in gynecological practice. These minimally invasive procedures are generally considered low risk; however, venous air/gas embolism is a potentially lethal complication. The reported incidence of hysteroscopy-related embolic events ranges from 10% to 50%, with mortality in severe cases reported up to 46% [1]. Accordingly, heightened risk awareness, early identification, appropriate management, and prevention are imperative for anesthesiologists and surgeons to avoid catastrophic outcomes. Herein, we report a case of venous air/gas embolism during operative hysteroscopy characterized by an unusual cardiovascular presentation of profound bradycardia followed by marked ST-segment elevation mimicking acute coronary syndrome. This case highlights the diagnostic value of rapid echocardiographic assessment and the importance of coordinated surgical and anesthetic vigilance for early recognition and management.

This case report has been reported in line with the SCARE (Surgical CAse REport) criteria [2].

## Case presentation

**Figure 1:**
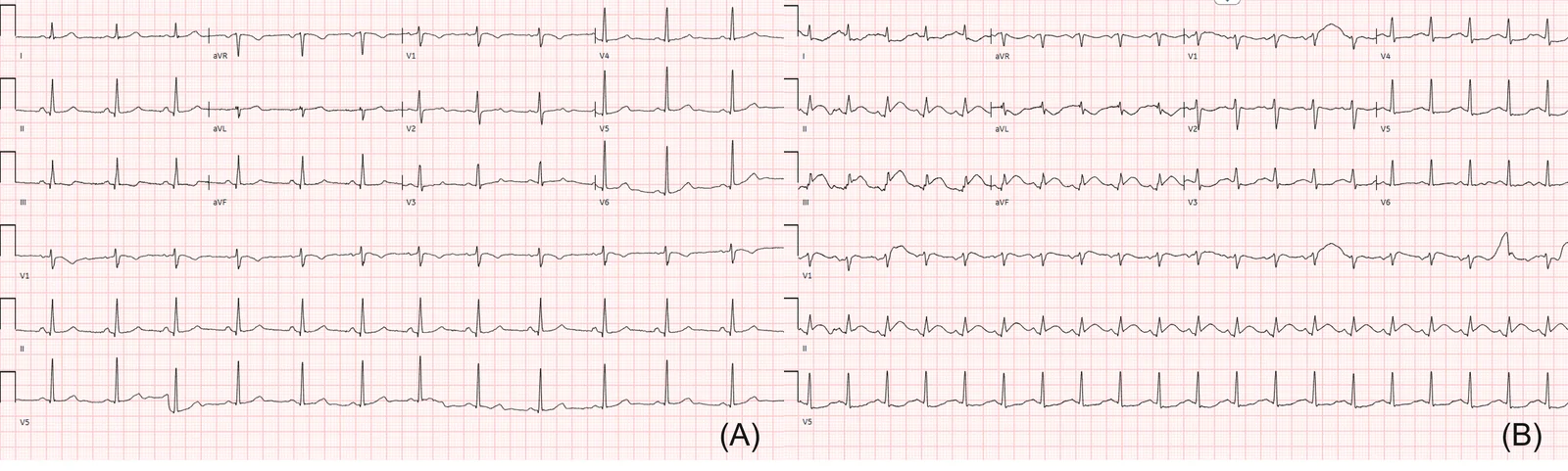
Comparison of preoperative and intraoperative electrocardiograms. (A) Preoperative electrocardiogram showing normal sinus rhythm without significant ST-segment abnormalities. (B) Intraoperative electrocardiogram obtained during the acute event showing marked ST-segment elevation in leads II, III, and aVF with reciprocal ST-segment depression in leads I and aVL.

**Figure 2:**
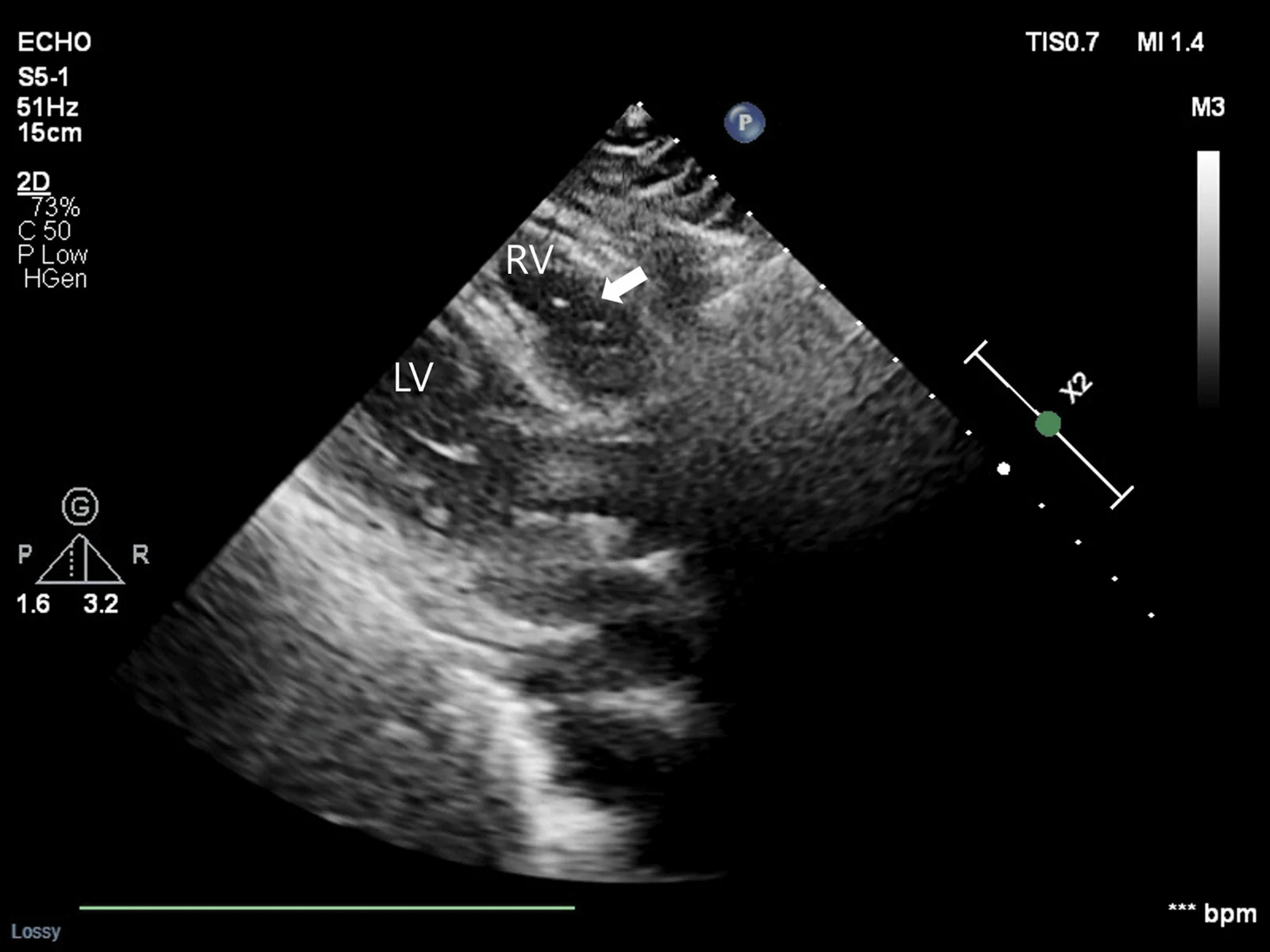
Transthoracic echocardiography demonstrating echogenic air bubbles within the right ventricle (white arrow) accompanied by transient right ventricular dysfunction.

A 46-year-old woman (height, 156 cm; weight, 50 kg) presented with dysmenorrhea, menstrual irregularity, and abdominal bloating. She reported a 12-month history of abdominal bloating and crampy dysmenorrhea with irregular menstrual cycles. The patient had a history of breast cancer treated with breast-conserving surgery and tamoxifen two years prior. She had no history of chronic cardiovascular, respiratory, or neurologic diseases and no known drug allergies. No significant personal or family history was reported. Abdominal examination revealed a soft, non-distended abdomen with mild lower quadrant tenderness and no rebound or guarding. Preoperative laboratory investigations, including serum electrolytes, renal and liver function tests, and coagulation profile, showed no clinically significant abnormalities. Mild anemia was present (hemoglobin, 10.8 g/dL). Preoperative electrocardiography showed normal sinus rhythm without significant ST-segment abnormalities (Figure 1A). General anesthesia was induced with propofol, remifentanil, and rocuronium, followed by endotracheal intubation. Mechanical ventilation was maintained in pressure-control volume-guaranteed mode with an inspired oxygen fraction of 0.5, tidal volume of 400 mL, and respiratory rate of 14 breaths/min. The patient was placed in the lithotomy position with a 20°-30° Trendelenburg tilt. Operative hysteroscopy was performed using bipolar electrosurgery with 0.9% normal saline as the uterine distension medium; no gas insufflation was used. The saline flow rate was 0.8 L/min. Intrauterine pressure was generally maintained at 60 mmHg and was temporarily increased up to 90 mmHg when necessary to obtain adequate visualization. Cervical dilation was performed using Hegar dilators, and a tenaculum was used. Repeated insertion and removal of hysteroscopic instruments were required during myoma resection. The exact fluid deficit could not be reliably determined from the available operative records. Approximately 50 min after the start of surgery, end-tidal CO₂ abruptly decreased from 32 to 24 mmHg, accompanied by bradycardia (heart rate 38 bpm) and hypotension (90/63 mmHg). Atropine 0.5 mg and ephedrine 4 mg were administered. Shortly thereafter, a portable 12-lead electrocardiogram revealed marked ST-segment elevation in the inferior leads (II, III, and aVF) with reciprocal changes in leads I and aVL, accompanied by tachycardia of 100-120 bpm (Figure 1B). Arterial blood gas analysis revealed severe metabolic acidosis (pH 7.011). Transthoracic echocardiography revealed echogenic air bubbles within the right ventricle accompanied by transient right ventricular dysfunction (Figure 2).

Transvaginal ultrasonography demonstrated about a 3.8 cm submucosal myoma on the uterine fundus distorting the endometrial cavity.

Following the abrupt decrease in PETCO₂ and development of hemodynamic instability, atropine and ephedrine were administered immediately. After initial stabilization, an arterial line was promptly inserted, and norepinephrine infusion was initiated for continuous hemodynamic support. Transthoracic echocardiography performed shortly thereafter demonstrated multiple echogenic air bubbles within the right ventricle. Because blood pressure and oxygenation improved rapidly with supportive management and 100% oxygen administration, additional rescue interventions, such as Durant positioning or central venous aspiration, were not performed. The procedure was discontinued, and the patient was transferred to the intensive care unit for further management.

The patient was extubated in the intensive care unit and stabilized with supportive care. Serial cardiac biomarker measurements demonstrated transient myocardial injury, with troponin I increasing from 0.057 ng/mL immediately after surgery to a peak value of 3.169 ng/mL on postoperative day 2, followed by a gradual decline. Creatine kinase-myocardial band (CK-MB) showed a similar transient elevation, and N-terminal pro-brain natriuretic peptide (NT-proBNP) was elevated during the acute phase (Table 1).

**Table 1:** Serial postoperative cardiac biomarker levels POD: postoperative day, CK-MB: creatine kinase-myocardial band, NT-proBNP: N-terminal pro-brain natriuretic peptide

Time point	Troponin I	CK-MB	NT-proBNP
Unit	ng/ml	ng/ml	pg/mL
Immediately after surgery	0.057	4.9	-
10 hours after surgery	1.653	23.6	621
POD2	3.169	24	-
POD3	1.111	2.5	-
POD4	0.175	<1.0	-
POD7	0.081	<1.0	-
One month after surgery	<0.010	<0.010	79.1
Reference range	0–0.04	0–4.7	0-121

The overall clinical and echocardiographic findings were considered consistent with stress-induced cardiomyopathy with right ventricular involvement, while coronary angiography demonstrated no luminal stenosis or evidence of obstructive coronary artery disease. The patient was discharged on postoperative day 7 without residual symptoms, with resolution of the electrocardiographic abnormalities and substantial improvement in cardiac biomarker levels. At the one-month follow-up, troponin I and CK-MB had normalized.

## Discussion

Surgical hysteroscopy is widely used for diagnostic and therapeutic purposes in gynecology. Complications include uterine perforation, infection, and venous air/gas embolism [3]. The risk of venous air/gas embolism is greater during operative than diagnostic hysteroscopy, and severe or fatal cases have been reported [4]. Procedures associated with venous air/gas embolism include intrauterine adhesiolysis, endometrial resection, and myomectomy [5].

Venous air/gas embolism during operative hysteroscopy can range from clinically silent or minimally apparent embolic events to more extensive gas embolism [6]. Air or gas entrained through exposed uterine vessels can partially obstruct the pulmonary vasculature, producing ventilation-perfusion mismatch and increased physiologic dead space, which in turn decreases PETCO_2_ and SpO_2_ (peripheral oxygen saturation) [7]. Large-volume emboli increase right ventricular outflow resistance and reduce pulmonary venous return, eventually leading to cardiovascular collapse [8]. Arterialization of emboli may arise from augmented right ventricular strain with passage to the left heart through a right-to-left shunt, resulting in serious neurologic and cardiac events [9]. Systemic embolism can also occur without an intracardiac shunt: an embolus traversing the pulmonary circulation may enter the left heart under high pulmonary arterial pressures, causing paradoxical air/gas embolism with coronary or cerebral occlusion [10]. Rademaker et al. reported paradoxical gas embolism during surgical hysteroscopy in a patient without an anatomic shunt [11]. Extensive testing (saline contrast test, Valsalva maneuver) did not reveal an intracardiac right-to-left shunt (e.g., septal defect, patent foramen ovale) or an extracardiac pathway (e.g., pulmonary arteriovenous malformation). They therefore proposed transpulmonary transport of numerous large bubbles that exceeded the lungs’ filtering capacity as the pathophysiologic mechanism [11].

Venous air and venous gas embolism are often conflated, but they should be considered separately. During hysteroscopic surgery, venous embolism can occur via damaged uterine vessels through several mechanisms: gas entry from electrocautery vapor, insufflated gases, such as carbon dioxide (CO_2_), or room air introduced during repeated instrument exchanges [6,7]. Because room air is less soluble than CO_2_, it poses a greater embolic risk [12]. Nevertheless, although CO_2_ is considered safe as a uterine distention medium when maintained at <100 mmHg and <100 mL/min, rapid intravascular influx of CO_2_ can be hazardous [13].

Prevention of venous air/gas embolism during operative hysteroscopy requires coordinated management by both the surgical and anesthesia teams. The uterine distension system should be carefully monitored, using the lowest intrauterine pressure that provides adequate visualization while avoiding unnecessary pressure gradients that may facilitate intravasation. All tubing and hysteroscopic instruments should be adequately de-aired, and entry of room air during instrument exchange should be minimized [14,15]. Cervical priming can facilitate atraumatic cervical entry, and a continuous drainage system should be maintained to refresh the distension medium and remove debris and bubbles [14]. Fluid deficit should be monitored throughout the procedure because the risk of fluid-related complications is associated with the volume of intravasated distension medium rather than the total volume infused [16,17]. Isotonic 0.9% NaCl is commonly used for uterine distension and avoids the electrolyte disturbances associated with hypotonic, electrolyte-free solutions; nevertheless, vigilance for fluid overload remains necessary [16]. The anesthesia team should remain aware of procedural factors, including the distension medium, intrauterine pressure, fluid deficit, duration of resection, and repeated instrumentation, and communicate promptly with the surgical team when abrupt capnographic or hemodynamic changes suggest possible venous air/gas entrainment.

In the present case, 0.9% normal saline was used as the uterine distension medium at a flow rate of 0.8 L/min, without gas insufflation. Intrauterine pressure was generally maintained at 60 mmHg and was temporarily increased up to 90 mmHg when necessary to obtain adequate visualization. The exact fluid deficit could not be reliably determined from the available operative records. Difficult and prolonged transcervical resection of the myoma, repeated instrument manipulation, and exposure of uterine venous channels may have facilitated air/gas entrainment. Although the exact source and composition of the embolic gas could not be definitively determined, entry of room air during instrument manipulation and electrosurgery-generated gas are possible mechanisms.

Previous reports have demonstrated that hysteroscopy-associated venous air/gas embolism can present with a wide clinical spectrum ranging from transient decreases in end-tidal CO₂ to catastrophic cardiovascular collapse. Reported risk factors include prolonged operative time, extensive endometrial resection or myomectomy, elevated intrauterine pressure, Trendelenburg positioning, repeated cervical instrumentation, and exposure of large venous channels during operative hysteroscopy [1]. Common intraoperative manifestations include abrupt reductions in PETCO₂, oxygen desaturation, hypotension, arrhythmias, electrocardiographic abnormalities, pulmonary edema, and cardiac arrest [1,8]. Diagnostic approaches described in the literature include transesophageal echocardiography, precordial Doppler ultrasonography, transthoracic echocardiography, arterial blood gas analysis, and characteristic capnographic changes [8]. Management strategies primarily focus on immediate discontinuation of surgery, prevention of further air entrainment, administration of 100% oxygen, hemodynamic support, patient repositioning, cardiopulmonary resuscitation when necessary, and postoperative intensive care monitoring [8]. Despite increased recognition of this complication, severe morbidity and fatal outcomes continue to be reported, emphasizing the importance of early diagnosis and multidisciplinary management.

In the present case, abrupt reduction of PETCO₂ was followed almost simultaneously by profound bradycardia, desaturation, hypotension, and marked ST-segment elevation. Although compensatory sympathetic tachycardia is more commonly described in venous air/gas embolism, bradycardia may occur through the Bezold-Jarisch reflex, particularly in severe embolic events associated with markedly reduced ventricular filling. The immediate onset of profound bradycardia without preceding tachycardia in this patient may therefore suggest a substantial embolic burden. In addition, marked ST-segment elevation and stress-induced cardiomyopathy with right ventricular involvement created a diagnostic challenge by mimicking acute coronary syndrome or primary cardiac pathology. Potential mechanisms include right ventricular ischemia and overload, paradoxical coronary embolization, and global myocardial ischemia secondary to hypoxia and hypotension. However, formal contrast-enhanced transthoracic echocardiography with a saline study or Valsalva maneuver was not performed; therefore, a small intracardiac right-to-left shunt could not be definitively excluded. Given the absence of overt echocardiographic evidence of a shunt and the presence of marked right ventricular dysfunction, right ventricular strain-related ischemia was considered the most plausible mechanism in this case. Although coronary gas embolism could not be definitively excluded, coronary angiography demonstrated no luminal stenosis or evidence of obstructive coronary artery disease, and the subsequent recovery of cardiac function favored stress-induced cardiomyopathy associated with severe right ventricular strain and hemodynamic instability.

Several clinically important lessons emerge from this case. First, venous air/gas embolism during hysteroscopic surgery may initially present with profound bradycardia rather than compensatory tachycardia, potentially reflecting severe embolic burden and vagally mediated cardiovascular collapse. Second, marked ST-segment elevation and transient stress-induced cardiomyopathy with right ventricular involvement may mimic acute coronary syndrome, creating significant diagnostic uncertainty during intraoperative hemodynamic deterioration. In this setting, rapid intraoperative transthoracic echocardiography was particularly valuable for promptly identifying intracardiac air bubbles and supporting early diagnosis. This case further emphasizes that life-threatening embolic complications can occur during operative hysteroscopy despite its generally minimally invasive nature. Therefore, vigilant monitoring, early recognition, and immediate multidisciplinary management remain essential for favorable clinical outcomes.

## Conclusions

Venous air/gas embolism should be considered when an abrupt decrease in end-tidal CO₂ is accompanied by hemodynamic or electrocardiographic abnormalities during operative hysteroscopy. In this case, profound bradycardia and marked ST-segment elevation mimicking acute coronary syndrome represented an unusual cardiovascular presentation. Prompt echocardiographic assessment facilitated the diagnosis by demonstrating intracardiac air bubbles and right ventricular dysfunction. Prevention and early recognition require close communication and coordinated vigilance between the surgical and anesthesia teams, with careful attention to the uterine distension system and procedural factors that may increase the risk of air/gas entrainment.
